# Ginkgolic Acid (GA) Inhibits the Growth of OCa by Inhibiting lncRNA MALAT1/JAK2 Axis

**DOI:** 10.1155/2021/5481271

**Published:** 2021-12-26

**Authors:** Zhiyi Fei, Yi Yu, Mi Xiang, Fang Luo

**Affiliations:** Department of Obstetrics and Gynecology, Wuhan Puren Hospital, Wuhan, China

## Abstract

**Objective:**

We aimed to observe the impact of ginkgolic acid (GA) on the proliferation and metastasis ability of ovarian cancer (OCa) cells and to further explore whether GA affects the malignant progress of OCa via regulating the lncRNA MALAT1/JAK2 axis.

**Methods:**

OCa cells SKOV3 and CAOV3 were administered with 1 ng/ml GA, 5 ng/ml GA, 10 ng/ml GA, 20 ng/ml GA, and DSMO as control, respectively. The cell proliferation and migration ability of the abovementioned cells in each group were measured by CCK-8 test and Transwell experiments. The expression levels of lncRNA MALAT1 and JAK2 protein were examined by qRT-PCR and western blot, respectively. Subsequently, in OCa cells treated with GA, lncRNA MALAT1 overexpression vector was transfected to continue to detect the proliferation activity and migration ability of each treatment group. Finally, the regulation of GA on activity of lncRNA MALAT1/JAK2 axis in OCa cells was further explored in nude mice.

**Results:**

Our data showed that the proliferation inhibition rate of cells at each ginkgolic acid concentration was higher than that of the control group (*P* < 0.05), suggesting that GA has an inhibitory influence on the proliferation of OCa cells, in a dose-dependent way. GA was able to inhibit the proliferation rate and migration ability of OCa cells. Administration of ginkgolic acid downregulated the levels of lncRNA MALAT1 and JAK2 protein. Overexpression of lncRNA MALAT1 partially reversed the inhibited OCa proliferative capacity caused by GA treatment. Consistent with the results observed *in vitro*, we also found that the OCa tumor weight and volume of nude mice injected with lncRNA MALAT1 overexpression vector were enhanced and JAK2 protein level increased remarkably in comparison to the ginkgolic acid group.

**Conclusions:**

In summary, GA may exert its inhibitory effect on the proliferative and migratory capacities of OCa cells through suppressing the activity of lncRNA MALAT1/JAK2 axis.

## 1. Introduction

Gynecological malignancy as the main cause of tumor occurrence and death in women globally mainly includes ovarian tumor, uterine tumor, fallopian tube tumor, vulvar tumor, and vaginal tumor, among which ovarian and uterus tumor are the most common [1, 2]. The incidence of OCa ranks second in female reproductive system malignancies, among which epithelial OCa is the most common histological type, accounting for about 90% of the total number of cases, and its mortality ranks first in gynecological tumors [3, 4]. The latest statistics from the National Cancer Institute show that the mortality rate of ovarian cancer has not changed significantly, and the 5-year survival rate is only 45.6%. Due to the lack of effective screening methods, 70–80% of patients have been in the advanced stage (stage III/IV) at the time of diagnosis, with a 5-year survival rate of only 20–30% [5, 6]. At present, tumor reduction surgery combined with platinum-based chemotherapy is the classic treatment for epithelial ovarian cancer. It has been clinically implemented for decades and is recognized as the most effective treatment for ovarian cancer worldwide; however, this classic approach has little effect on patients with platinum-resistant and recurrent ovarian cancer [7, 8]. Although the increase and improvement of treatment methods in recent years have enabled 80% of patients to achieve clinical remission, more than 60% of patients will eventually develop tumor recurrence, metastasis, and drug resistance, and the treatment effect of recurrent and drug-resistant OCa still remains to be solved [9, 10]. Early detection, diagnosis, and treatment of OCa are key factors affecting the prognosis of OCa. Thus, it is necessary to further explore the mechanism of the occurrence and development of OCa and drug resistance to improve OCa patients' prognosis [11].

Ginkgolic acid (GA) is a derivative of 6-alkyl or 6-enyl salicylic acid. The number of side chain carbon atoms on the six bits can range from 13 to 19, and the number of side chain double bonds can range from 0 to 2. It can be seen that GA is a mixture composed of homologues of side chains of different lengths [12, 13]. Studies have shown that GA has a good antitumor effect and can inhibit the growth of a variety of tumor cells *in vitro*; while *in vivo*, it can also reduce the volume of mouse sarcoma and prolong the survival of mice [14, 15]. However, it is not clear whether ginkgolic acid can inhibit the malignant progression of OCa and how it regulates the progression of tumor cells [16, 17]. LncRNA plays a crucial role in carcinogenesis, invasion, and metabolism of many tissues [17, 18]. Therefore, this study mainly discussed the proliferation, metastasis, and molecular expression of ginkgolic acid in human OCa cells to further investigate the effect of ginkgolic acid on malignant progression of OCa and its underlying mechanism.

## 2. Methods

### 2.1. Cell Lines and Reagents

Human-derived OCa cells (SKOV3 and CAOV3) provided by the American Type Culture Collection (ATCC) (Manassas, VA, USA) company were cultured in Dulbecco's modified eagle medium (DMEM) supplemented with 10% fetal bovine serum (FBS) (Gibco, Rockville, MD, USA) in an incubator with 5% CO_2_ at 37°C.

### 2.2. Transfection

Transfection was performed with pcDNA3.1-NC and pcDNA3.1-MALAT1 according to the manufacturer's instructions when cell density reached 30–50%. 48 hr later, cells were collected for cell function experiments.

### 2.3. Cell Counting Kit-8 (CCK-8) Assay

Cells of each group were seeded in a 96-well plate at a density of 2500 cells/well. CCK-8 assay (Dojindo, Kumamoto, Japan) was conducted based on instructions.

### 2.4. Transwell Assay

Each treated OCa cells SKOV3 and CAOV3 were resuspended in serum-free medium and counted. SKOV3 cells (4 × 104 cells) or CAOV3 cells (2 × 104 cells) were suspended in 200 *μ*l serum-free MEM medium. The cells were then seeded into the upper chamber, and 500 *μ*l of DMEM medium with 10% serum was added to the bottom chamber. After culturing in a cell incubator for 24 hours, the cells in the upper chamber were removed with a cotton swab; the lower cells were fixed with 4% paraformaldehyde for 30 minutes and then stained with crystal violet for 30 minutes. The cell membrane was washed with phosphate buffered saline (PBS), and the cells were photographed and counted under a 200-fold upright microscope.

### 2.5. QPCR

After corresponding treatment of OCa cells SKOV3 and CAOV3, 1 ml of TRIzol was used to lyse the cells to extract total RNA. QPCR detection was implemented based on the instructions of SYBR® Premix Ex Taq™ Kit (TaKaRa, Tokyo, Japan), with glyceraldehyde 3-phosphate dehydrogenase (GAPDH) and U6 as internal parameters. Primers used in the qPCR reaction were as follows: lncRNA MALAT1: forward: 5′-GCTCTGTGGTGTGGGATTGA-3′, reverse: 5′-GTGGCAAAATGGCGGACTTT-3'; GAPDH: forward: 5′-CCTGGCACCCAGCACAAT-3′, reverse: 5′-GCTGATCCACATCTGCTGGAA-3'.

### 2.6. Western Blot

Western blot analysis was performed according to standard procedures. The primary antibodies against JAK2 (Dilution: 1/500; CatNOs: ab108596) and GAPDH (dilution: 1 : 500; CatNOs: ab37168), and the secondary antibodies (dilution: 1/2000; CatNOs: ab6721) were all purchased from Abcam (Cambridge, MA, USA).

### 2.7. *In Vivo* Xenograft Model

*In vivo* nude mice tumorigenesis experiments were approved by The Animal Ethics and Use Committee. Fifteen 8-week-old male nude mice were purchased from the animal center and randomly divided into 3 groups (5 in each group). The OCa cells treated with GA were transfected with the MALAT1 overexpression vector and injected into the axilla of the mice subcutaneously. The tumor size was monitored every 5 days; then, the mice were sacrificed after 30 days. The volume of all samples is calculated using the following formula: tumor volume = (width 2 × length)/2.

### 2.8. Statistical Analysis

Continuous variables were analyzed using Student's *t*-test, and categorical variables were analyzed using *χ*^2^ test or Fisher's exact probability method. Data were processed by Statistical Product and Service Solutions (SPSS) 22.0 program (IBM, Armonk, NY, USA) and were expressed as *X* ± SD. *P* < 0.05 was considered statistically significant.

## 3. Results

### 3.1. GA Inhibits OCa Cell Proliferation and Migration

**Figure 1:**
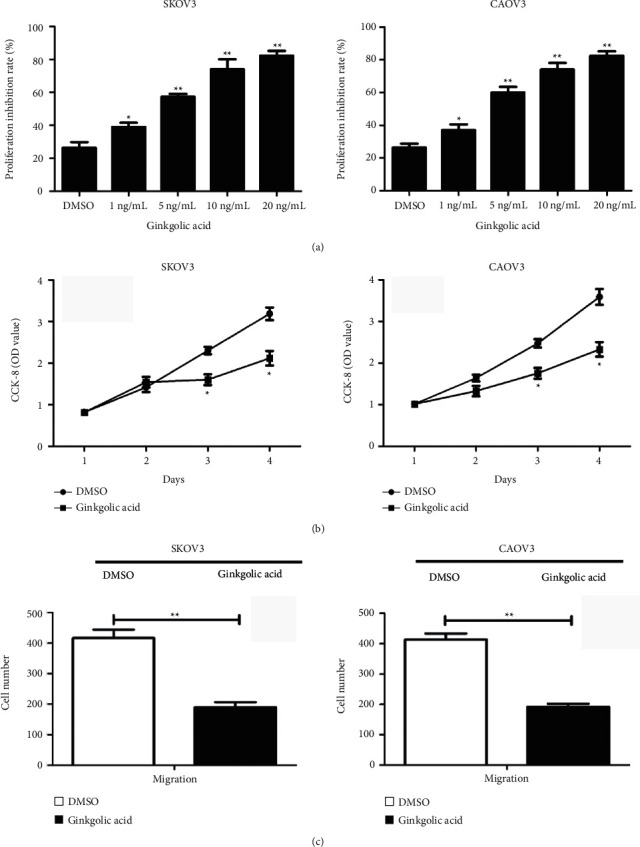
Ginkgolic acid inhibits the proliferation and migration of ovarian cancer cells. (a) Ginkgolic acid inhibited cell proliferation in a dose-dependent manner (1 ng/ml, 5 ng/ml, 10 ng/ml, and 20 ng/ml) in ovarian cancer cell lines SKOV3 and CAOV3; (b) ginkgolic acid inhibited cell proliferation in a time-dependent manner (1 d, 2 d, 3 d, and 4 d) in ovarian cancer cell lines SKOV3 and CAOV3; (c) Transwell test was used to detect the effect of ginkgolic acid treatment on cell migration in ovarian cancer cell lines SKOV3 and CAOV3. Data are presented as average ± SD, ^*∗*^*P* < 0.05, ^*∗∗*^*P* < 0.01.

The proliferation inhibition rate of OCa cells in each treatment concentration of GA treatment group was higher than that of the control group (*P* < 0.05), and the inhibitory effect was enhanced as the concentration of GA increases (Figure 1(a)). Meanwhile, Figure 1(b) shows that the OD value of OCa cells treated with GA observed in CCK-8 test decreased in comparison to the DMSO (0.1%) control group, suggesting an attenuated proliferative ability induced by GA (Figure 1(b)). Consistent to the changes in cell proliferation, the migration capacity of OCa cells was also attenuated by treatment of GA (Figure 1(c)).

### 3.2. Ginkgolic Acid Regulates lncRNA MALAT1 Expression-JAK2 Axis of OCa Cells

**Figure 2:**
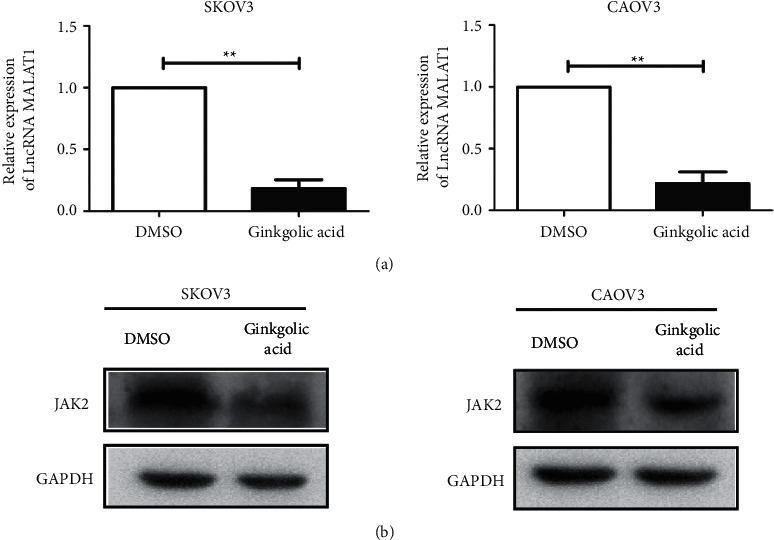
Ginkgolic acid regulates the lncRNA MALAT1-JAK2 axis. (a) qRT-PCR experiment was used to detect the expression level of lncRNA MALAT1 in ovarian cancer cell lines SKOV3 and CAOV3 after ginkgolic acid treatment; (b) western blot test was used to detect the expression level of JAK2 in ovarian cancer cell lines SKOV3 and CAOV3 after ginkgolic acid treatment. Data are presented as average ± SD, ^*∗∗*^*P* < 0.01.

To test the mechanism by which GA inhibits the malignant progression of OCa, we examined the expression of lncRNA MALAT1 and JAK2 protein in OCa cells after GA treatment. As a result, we found a reduction in both the mRNA expression of lncRNA MALAT1 (Figure 2(a)) and the protein level of JAK2 (Figure 2(b)).

### 3.3. lncRNA MALAT1 Promotes the Migration as Well as Proliferation of OCa Cells Treated with GA

**Figure 3:**
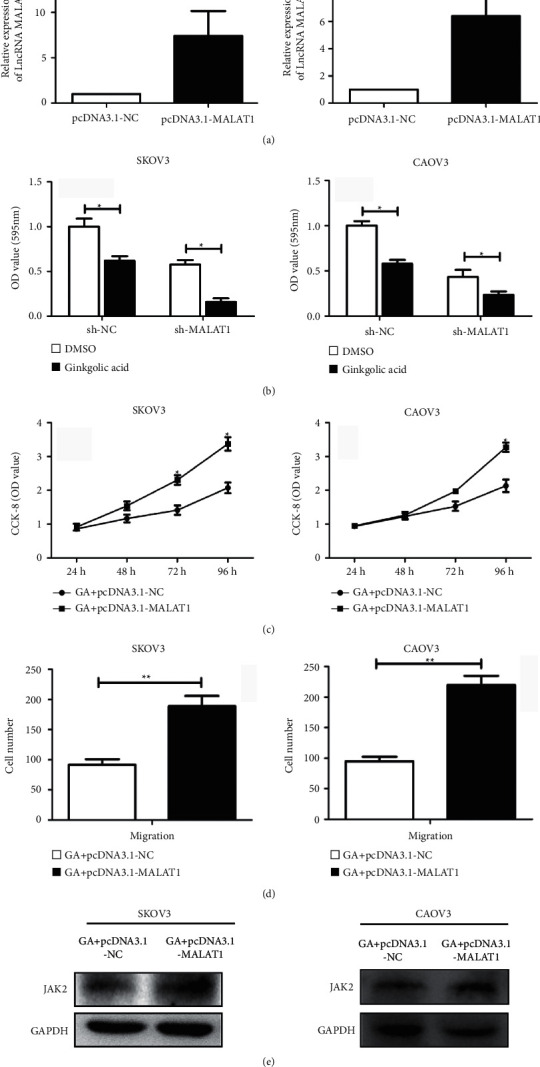
LncRNA MALAT1 promotes the proliferation and migration of ovarian cancer cells. (a) The qRT-PCR experiment detected the transfection efficiency of ovarian cancer cell lines SKOV3 and CAOV3 after transfecting lncRNA MALAT1 overexpression vector; (b) knocking down lncRNA MALAT1 enhanced the inhibitory impact of GA on OCa cells proliferation; (c) overexpression of lncRNA MALAT1 enhanced cell viability; (d) overexpression of lncRNA MALAT1 enhanced cell migration; (e) western blot test was used to detect the expression level of JAK2 after transfection of lncRNA MALAT1 overexpression vector in ovarian cancer cell lines SKOV3 and CAOV3 after ginkgolic acid treatment. Data are presented as average ± SD, ^*∗*^*P* < 0.05, ^*∗∗*^*P* < 0.01.

To further explore the role of lncRNA MALAT1 in the function of OCa cells, we constructed lncRNA MALAT1 overexpression model and verified the transfection efficiency by qPCR experiment (Figure 3(a)). It was found that knocking down lncRNA MALAT1 enhanced the inhibitory impact of GA on OCa cells proliferation (Figure 3(b)), while overexpression of lncRNA MALAT1 partially counteracted that (Figure 3(c)), measured by CCK-8 test. At the same time, Transwell experiments also showed that the number of transferring cells in Transwell increased remarkably after overexpression of lncRNA MALAT1, suggesting an enhanced metastasis (Figure 3(d)). We also observed an increased protein level of JAK2 induced by lncRNA MALAT1 upregulation, revealed by western blot assay (Figure 3(e)).

### 3.4. Ginkgolic Acid Inhibits Tumorigenic Ability of Nude Mice with OCa

**Figure 4:**
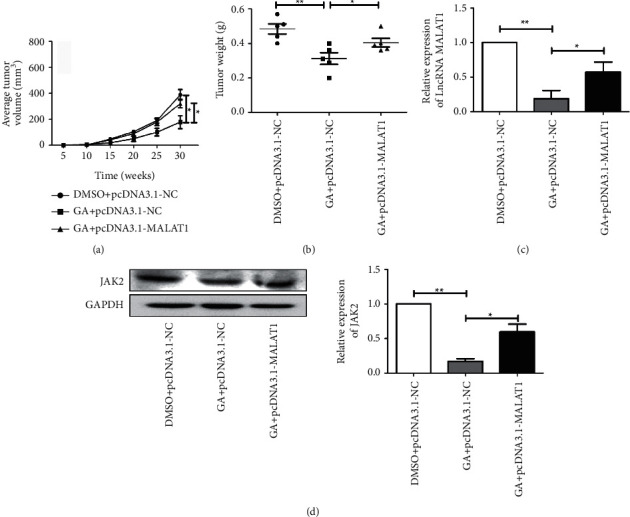
Ginkgolic acid inhibits the tumorigenic ability. (a). Ginkgolic acid inhibited the tumor volume, while pcDNA3.1-MALAT1 enhanced the tumor volume; (b) ginkgolic acid inhibited the tumor weight, while pcDNA3.1-MALAT1 enhanced the tumor weight; (c) the expression of lncRNA MALAT1 was remarkably reduced by ginkgolic acid; (d) the protein expression of JAK2 was significantly inhibited by ginkgolic acid, however, was partially reversed after lncRNA MALAT1 overexpression. Data are presented as average ± SD, ^*∗*^*P* < 0.05, ^*∗∗*^*P* < 0.01.

OCa SKOV3 cells were inoculated into each nude mouse and injected in the left armpit; all of the mice were then treated with GA. As expected, compared with the blank control group, the tumor volume and weight of nude mice treated with GA were remarkably reduced, but could be enhanced partially by transfection of pcDNA3.1-MALAT1 (Figures 4(a) and 4(b)). Subsequently, total RNA and protein of nude mice tumor tissues were extracted, and qPCR and western blot experiments were carried out to measure the level of lncRNA MALAT1 and JAK2 protein. Figures 4(c) and 4(d) show that the expressions of JAK2 protein and lncRNA MALAT1 were both remarkably reduced in nude mice treated with GA, however, could be partially reversed after lncRNA MALAT1 overexpression vector was injected in. Taken together, the above observations of *in vivo* experiments demonstrate that GA may suppress the tumorigenic ability of OCa cells in nude mice by downregulating the lncRNA MALAT1/JAK2 axis.

## 4. Discussion

Ovarian cancer is the most fatal gynecological malignancy and the second leading cause of cancer death in women [1–3]. The current standard treatment for advanced ovarian cancer includes surgery combined with radiotherapy and chemotherapy [4–7]. Despite the continuous development of new therapies (such as targeted therapy and immunotherapy) and the improvement of the 5-year survival rate [7, 8], the inherent or acquired multidrug resistance remains a major challenge [8–10]. Currently, no biomarkers for predicting chemotherapy-induced reactions have been used in clinical practice [9–11].

Ginkgolic acid has obvious growth inhibition effect on a variety of tumor cell lines *in vitro* [12, 13]. Some studies reported that ginkgolic acid compound was extracted from the outer seed skin of *Ginkgo biloba*, which had inhibitory effects on the growth of HCT-15 colorectal cancer cell line [12, 13], MCF-7 breast cancer cell line, A-549 lung cancer cell line, HT-1197 bladder cancer cell line, and SKOV3 uterine cancer cell line, but had little cytotoxicity to normal intestinal cells [12–15]. The present data revealed that the proliferation inhibition rate of OCa cells treated with ginkgolic acid at each concentration was higher than that of the control group, and the metastasis and proliferative ability of OCa cells were remarkably reduced by ginkgolic acid treatment.

At present, studies have found that LncRNA, as an important part of gene regulation network, has important significance in biological behaviors such as cell proliferation, differentiation, apoptosis, invasion, and metastasis [16, 17]. Therefore, to find out whether the abnormal expression of lncRNA in OCa is related to the antitumor properties of ginkgolic acid and to analyze its function will help to improve the diagnosis and treatment level of OCa and improve the quality of life of patients. We found that the mRNA expression level of lncRNA MALAT1 and the protein expression of JAK2 were both remarkably downregulated after treatment of ginkgolic acid. To further explore the regulation of ginkgolic acid on lncRNA MALAT1 expression-JAK2 axis in OCa cells, we have constructed a MALAT1 overexpression model by lentivirus. As a result, we demonstrated that overexpression of lncRNA MALAT1 could promote the proliferative capacity and migration of OCa treated with ginkgolic acid and enhanced the protein levels of JAK2 in the abovementioned cells. Additionally, tumor formation in nude mice also demonstrated that ginkgolic acid inhibits tumor formation in nude mice by downregulating the lncRNA MALAT1/JAK2 axis.

## 5. Conclusions

In summary, these evidences suggest that ginkgolic acid inhibits the proliferation and migration of OCa cells through the downregulation of lncRNA MALAT1 expression/JAK2 axis. However, further molecular mechanism should still be explored in our future study. With the deepening of research, further understanding of ginkgolic acid inhibition of lncRNA MALAT1/JAK2 axis on the biological function of OCa cells and its role in the process of tumor development will be more conducive to the diagnosis, treatment, and prognosis assessment of OCa.

## Data Availability

The datasets used and analyzed during the current study are available from the corresponding author on reasonable request.
